# HIV Nef and Vpu protect HIV-infected CD4+ T cells from antibody-mediated cell lysis through down-modulation of CD4 and BST2

**DOI:** 10.1186/1742-4690-11-15

**Published:** 2014-02-06

**Authors:** Tram NQ Pham, Sabelo Lukhele, Fadi Hajjar, Jean-Pierre Routy, Éric A Cohen

**Affiliations:** 1 Laboratory of Human Retrovirology, Institut de Recherches Cliniques de Montréal (IRCM), 110 Pine Avenue West, Montreal H2W 1R7, Quebec, Canada; 2 Division of Hematology and Chronic Viral Illness Service, Royal Victoria Hospital, McGill University Health Centre, Montreal H3A 1A1, Quebec, Canada; 3 Department of Microbiology, Infectiology and Immunology, Université de Montréal, Montréal H3T 1J4 Québec, Canada

**Keywords:** HIV accessory proteins Nef and Vpu, BST2, CD4-Env interactions, ADCC

## Abstract

**Background:**

HIV proteins Nef and Vpu down-modulate various host factors to evade immune defenses. Indeed, the CD4 receptor is down-regulated by Nef and Vpu, whereas virion-tethering BST2 is depleted by Vpu. Antibody-dependent cell-mediated cytotoxicity (ADCC) is increasingly recognized as a potentially powerful anti-HIV response. Given that epitopes which are specific for ADCC-competent anti-HIV antibodies are transitionally exposed upon CD4-mediated HIV entry, we investigated whether by depleting CD4 and BST2, HIV could negatively affect ADCC function.

**Results:**

Using anti-envelope (Env) Abs A32 and 2G12 to trigger ADCC activity, we find that interactions between CD4 and Env within infected cells expose ADCC-targeted epitopes on cell-surface Env molecules, marking infected T cells for lysis by immune cells. We also provide evidence to show that by cross-linking nascent virions at the plasma membrane, hence increasing cell-surface Env density, BST2 further enhances the efficiency of this antiviral process. The heightened susceptibility of T cells infected with a virus lacking Nef and Vpu to ADCC was recapitulated when plasmas from HIV-infected patients were used as an alternative source of Abs.

**Conclusions:**

Our data unveil a mechanism by which HIV Nef and Vpu function synergistically to protect infected cells from ADCC and promote viral persistence. These findings also renew the potential practical relevance of ADCC function in vivo.

## Background

The human immunodeficiency virus (HIV)-type 1 gains access to its target cells, primarily CD4+ T cells and macrophages, through the cellular receptor CD4 and co-receptor CCR5 or CXCR4. The HIV-1 RNA genome encodes many proteins including the structural envelope (Env) and accessory proteins Vpu and Nef. Over the years, studies have been conducted to decipher how HIV-1 exploits its viral elements, especially the accessory proteins, to modulate the host’s fundamental cellular machineries and responses in order to perpetuate its existence. Aside from their involvement in altering the expression of other host factors, Vpu and Nef, along with Env, can down-regulate CD4, albeit through distinct mechanisms [1,2]. Indeed, Env and Vpu, which are expressed late during viral replication, target newly synthesized CD4 molecules in the endoplasmic reticulum (ER), while the early expressed Nef focuses on CD4 already at the plasma membrane (PM). CD4 down-modulation by Env [3,4] is mediated through the formation of CD4-Env complexes in the ER, thus preventing CD4 trafficking to the cell surface [5], whereas that by Vpu occurs through CD4 ubiquitination and degradation via an ER-associated protein degradation (ERAD)-like mechanism [6,7]. Nef induces endocytosis of cell-surface CD4 molecules, targeting them for degradation in the lysosomes [8,9]. This functional redundancy leading to CD4 depletion is likely to be beneficial to the virus since it is thought to allow efficient Env trafficking to viral assembly sites while at the same time preventing superinfection and premature cell death [2,10]. In addition, Vpu also down-modulates BST2, a type 1 interferon-induced host factor that cross-links nascent virions at the cell surface, thus restricting their release from infected cells [11,12].

Antibody-dependent cell-mediated cytotoxicity (ADCC) is a type of humoral immune response mediated by effector cells of the innate immune system including natural killer (NK) cells and monocytes/macrophages [13]. In ADCC, binding of antibodies (Abs) to antigens present on target cells occurs through the Fab portion of the Ab, and leads to target-effector cell engagement via the Fc portion of the Ab and the Fcγ receptor (FcR) on effector cells. Binding of antigen-coated IgG to the FcR presumably causes a release of cytolytic granules and subsequent lysis of target cells.

Over the past decade, Fc-mediated effector functions including ADCC have been increasingly recognized as a potentially powerful host response against HIV-1 infection and dissemination. Indeed, findings from studies with HIV-infected patients and simian immunodeficiency virus (SIV)-infected macaques have implicated ADCC as an immune correlate of viral selection pressure, dampened viral replication, delayed disease progression, and even infection immunity [14-17]. Along this line, non-neutralizing Abs that can mediate FcR-dependent effector functions against HIV-1 [18-20] are thought to have contributed to protection in the Thai RV1144 vaccine trial [21]. Furthermore, passive transfer of Abs to macaques established the critical importance of the Fc portion of IgGs in preventing infection [22,23]. In summary, these studies underscore the often underappreciated importance of Fc-mediated effector responses driven by non-neutralizing Abs in the overall Ab-mediated protection against HIV-1.

The strength of ADCC function can be influenced by various host and viral factors. Among these are: (1) FcR expression levels on effector cells, (2) specificity of the Fab region, (3) abundance of viral antigens, and (4) accessibility of Abs to their cognate epitopes. In this context, recent studies focused on advancing our understanding of the nature of Abs that are capable of directing lysis of infected T cells [18,19,24]. As such, much of the research has been centered on Abs against the HIV-1 Env, since this protein represents a major viral antigen targeted by the host’s immune responses. Env is expressed on the surface of infected cells and is incorporated into virions during viral assembly. The functional spike of the virion is a trimeric complex consisting of gp120 and the non-covalently bound transmembrane gp41 subunit [25]. Env gp120 is exposed on the virion surface and binds to the CD4 receptor, whereas gp41 is normally buried within the viral envelope. Upon binding to CD4, gp120 undergoes sequential conformational changes that allow interactions with one of the primary co-receptors, CXCR4 or CCR5, which subsequently trigger exposure of the fusogenic gp41 ectodomain within the Env trimer [26]. Ultimately, the transition of the gp41 ectodomain configuration into a six-helix bundle results in fusion of the virus to target cells [26].

Most recently, it has been suggested that the face of gp120 occluded in the trimeric Env by gp41 is a potent ADCC target [24]. Indeed, analyses of human monoclonal Abs that recognize transitional epitopes exposed during Env-CD4 interactions revealed a strong bias of ADCC-competent Abs for Cluster A epitopes contained within this region of gp120 [24]. Among such anti-Env Abs is A32, which recognizes a discontinuous epitope on the inner domain of gp120, and has been documented to be capable of mediating ADCC [18,19]. The A32 epitope, which is expressed on CD4+ T cells infected *in vitro* with transmitted/founder viruses, could trigger efficient ADCC activity on both virally infected and gp120-coated CD4+ T cells [18]. More importantly, the A32 Fab fragment could block the majority of ADCC activity in plasma of HIV-1 infected patients, suggesting that if efficiently accessible, the A32 epitope is highly recognizable by Abs produced during HIV infection [18].

In light of the data discussed above, we asked whether HIV might exploit its natural propensity to down-modulate CD4 and BST2 to conceal ADCC-targeted epitopes and shield infected cells from destruction through ADCC. Here-in, using an *in vitro* infection system whereby primary CD4+ T cells are infected with isogenic viruses deficient of Nef and/or Vpu accessory proteins, we delineate the synergistic contributions of these two HIV proteins to the removal of CD4 and BST2 from the cell surface, thereby shielding infected T cells from ADCC. With these results, our study unveils a potential mechanism by which HIV evades the host’s immune defenses to promote persistence.

## Results

### Enhanced binding of anti-Env antibodies on CD4+ T cells infected with viruses deficient of HIV Nef and/or Vpu

**Figure 1:**
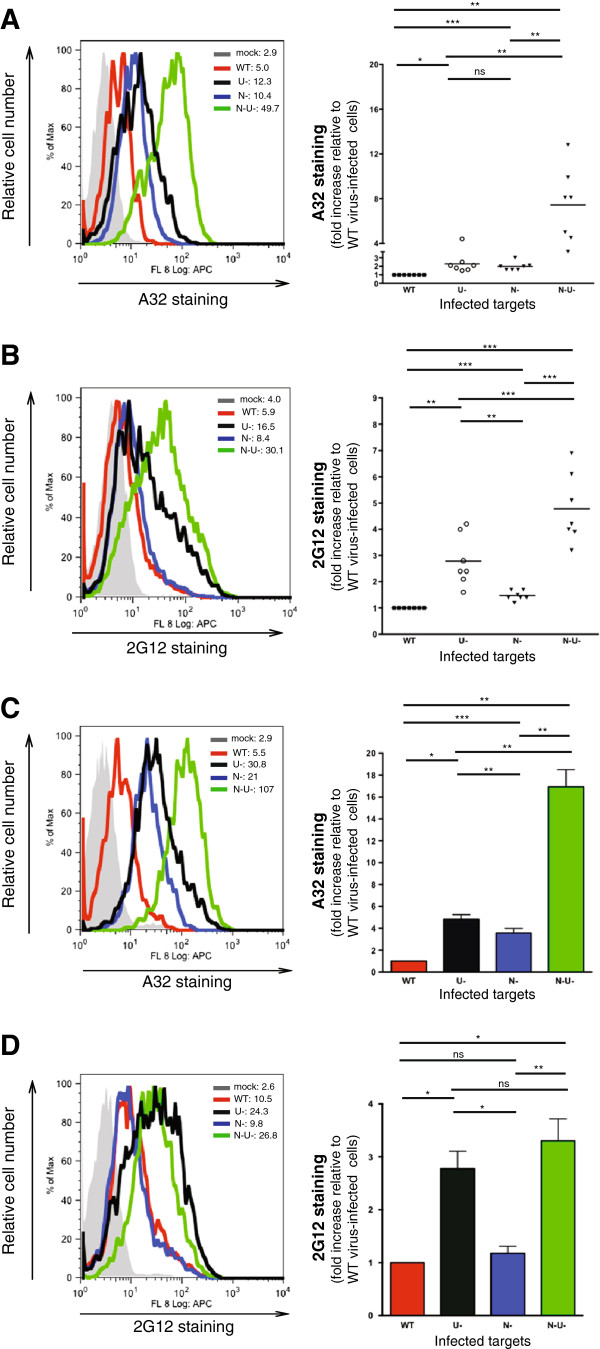
**HIV-1 envelope expression profiles on infected CD4+ T cells.** CEM.NKR **(A and B)** or activated primary CD4+ T cells **(C and D)** were infected with CCR5-tropic NL4.3.ADA.IRES.GFP wild-type (WT) virus or derivatives lacking Vpu (U-), Nef (N-) or both (N-U-) as detailed in Methods. Infected cells were analyzed by flow cytometry for Env expression using anti-Env A32 and 2G12 mAbs. **(A-D)** The left panels depict the extent of Env staining shown as geo-mean fluorescence intensity (MFI) on gated GFP^+^ cells from a representative infection. The right panels summarize the results of **(A and B)** seven experiments with each dot representing an analysis, and of **(C and D)** data obtained with primary CD4+ T cells from four donors. Shown are average fold increase (+/- SEM) in Env staining relative to WT virus-infected cells. Fold increase was determined as the ratio of MFIs of GFP^+^ cells infected with different mutants over that for the WT virus. Statistical analysis of data was done using paired Student’s t-tests.

To assess the recognition of Env by anti-Env Abs on infected T cells, CD4+ T cells were infected with CCR5-tropic NL4-3.ADA.IRES.GFP WT virus or its derivatives lacking Vpu (∆Vpu or U-), Nef (∆Nef or N-) or both (∆Nef∆Vpu or N-U-) and evaluated for Env expression. For a comparative analysis with A32, we used neutralizing Ab 2G12, which recognizes a discontinuous, glycan-dependent epitope on the gp120 outer domain and, as such, is distinct from other neutralizing Abs that recognize CD4-induced epitopes [27]. To this end, Env staining by A32 was about 2 to 2.5-fold higher on CEM.NKR CD4+ T cells infected with the ∆Nef or ∆Vpu virus and intriguingly, nearly 8-fold higher on those infected with the ∆Nef∆Vpu virus (P < 0.005) (Figure 1A). Notably, the Env staining profile by 2G12 was different with the ∆Vpu virus relative to the ∆Nef in that the former displayed a significantly higher increase in epitope recognition (P < 0.005), suggesting a potential contribution of BST2 to this enhancement. Similar to A32, 2G12 staining was significantly higher with the ∆Nef∆Vpu virus (P < 0.0005) (Figure 1B). The Env staining patterns by A32 and 2G12 were largely similar for primary CD4+ T cells infected with the same viruses, indicating that the data were not unique to cell lines (Figure 1C and D).

### Development and validation of a FACS-based ADCC assay

**Figure 2:**
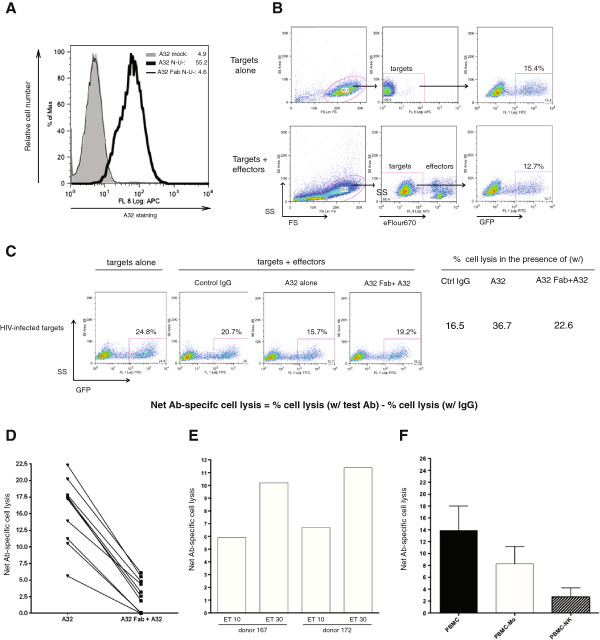
**Antibody-dependent cellular cytotoxicity assay: development and validation.** CEM.NKR cells were infected for 48 h with CCR5-tropic NL4.3.ADA.IRES.GFP ∆Nef∆Vpu and analyzed for their susceptibility to ADCC by PBMC under different testing parameters. Target cells were exposed to either control IgG, A32 or, alternatively, pre-exposed to the A32 Fab prior to the A32 exposure. Env staining was done as described in Figure 1 legend and ADCC was performed as described in Methods. **(A)** Evaluation of A32 staining specificity using the A32 Fab. **(B)** Gating strategy to select eFlour670-negative, GFP + target cells by flow cytometry. **(C)** Determination of net Ab-specific cell lysis using gating strategy depicted in **(B)**. The number shown inside dot plot depicts % of GFP^+^ cells remaining at the end of assay. Percent of cell lysis was calculated as [(% GFP^+^ cells in the absence of effectors -% GFP^+^ cells in the presence of effectors plus control IgG, A32 or A32 Fab + A32)/% GFP^+^ cells in the absence of effectors] × 100. **(D)** Cell lysis in the absence (A32) or presence of the A32 Fab fragment (A32 Fab + A32). Net Ab-specific cell lysis was obtained following subtraction of % cell lysis in the presence of IgG from that by either A32 or A32 Fab + A32. Each line represents PBMC-mediated lysis from a donor. **(E)** Effect of varying ET ratios (10 or 30) on net Ab-specific cell lysis using A32 as test Ab and IgG as control. Shown are results from two representative donors. **(F)** Contributions of different cell subsets to induction of ADCC. Total PBMC or PBMC depleted of monocytes/macrophages (PBMC-Mo) or NK cells (PBMC-NK) were used as effector cells. A32 was used as test Ab and IgG as control. Histograms represent average net A32-specific lysis +/- SD of four experiments with five donors.

As shown in Figure 2A, enhanced A32 binding on infected T cells could be blocked by pre-incubating target cells with the A32 Fab fragment prior to A32 exposure, demonstrating its specificity. Subsequently, we examined whether augmented binding of A32 on T cells would promote their lysis by ADCC.

Our FACS-based ADCC assay makes use of the fact that infected target cells were GFP-marked, while effector cells were labelled with a dye, allowing for subsequent gating of GFP-positive/dye-negative cells at the end of the assay (Figure 2B). The % cell lysis was determined by [(proportion of GFP^+^ cells in the absence of effector cells – proportion of GFP^+^ cells in the presence of effector cells and test Ab)/proportion of GFP^+^ cells in the absence of effector cells] × 100. For the example shown in Figure 2C, % cell lysis in the presence of A32 was computed to be [(24.8-15.7)/24.8] × 100 = 36.7%. Similarly, % cell lysis in the presence of control IgG was 16.5%. Importantly, in all evaluations, % cell lysis mediated by control IgG was determined simultaneously with test Abs, and used as background killing to calculate the net Ab (e.g., A32)-specific cell lysis. For Figure 2C example, the net A32-mediated lysis was calculated to be 36.7%-16.5% = 20.2%. By the same formulas, the net A32-mediated lysis in the presence of the A32 Fab was 6.1% (22.6%-16.5%). The attenuated ADCC activity, as a result of target cell pre-treatment with the A32 Fab, was replicated over many analyses using PBMC from different donors as effectors (Figure 2D). In addition, Figure 2D also revealed a differential ability by different PBMC donors to induce ADCC. The observed variations, which are not uncommon in analyses involving primary cells, could be due to inter-individual differences in FcR expression levels or the activation status of effector cells at the time of PBMC acquisition.

To further validate the assay, we examined the extent of target cell lysis using different effector:target (ET) ratios, and found that the magnitude of ADCC was ET-dependent (Figure 2E). Lastly, to determine which immune cell subsets might be responsible for inducing cell death, we used, as effector cells, total PBMC or PBMC that had been depleted of NK cells or monocytes/macrophages. As shown in Figure 2F, both cell subsets could elicit ADCC, although NK cells were apparently more efficient.

### Heightened susceptibility of CD4+ T cells infected with Nef- and Vpu- deficient HIV to A32-mediated ADCC

**Figure 3:**
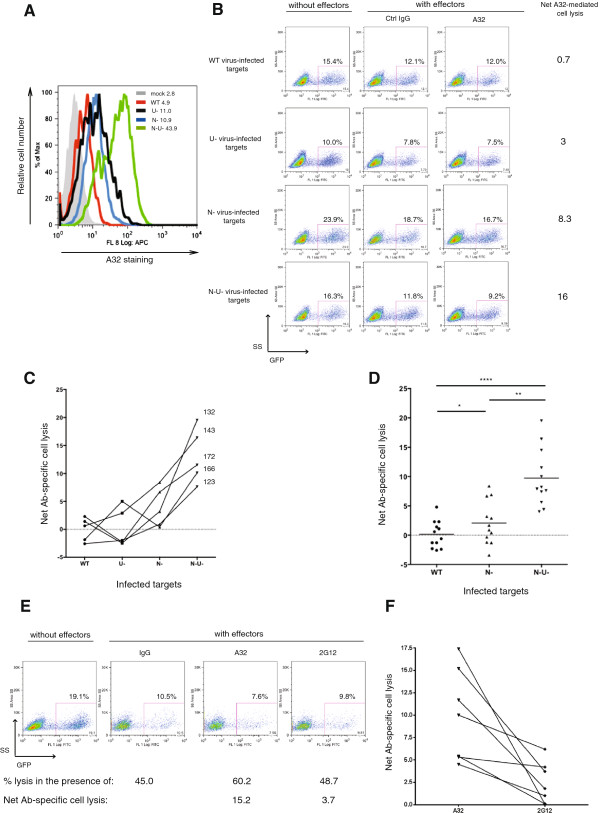
**Nef and Vpu reduce susceptibility of infected CD4+ T cells to A32-mediated ADCC.** CEM.NKR cells were infected with CCR5-tropic NL4.3.ADA. IRES.GFP viruses as mentioned in Figure 1 legend. Target cells were incubated with control IgG, A32 or 2G12 and analyzed for susceptibility to ADCC mediated by PBMC effector cells. Net Ab-specific cell lysis was computed as described in Figure 2 legend. As a qualitative control for infection, prior to the ADCC evaluation, target cells were also stained for Env expression using anti-Env Abs. **(A)** Env staining of target cells using A32. **(B)** An example of net Ab-specific cell lysis (using A32 as test Ab) from a representative donor (donor 143 in Panel **C**). **(C)** Comparative analysis of susceptibility of different target cells to A32-mediated ADCC. The five sets of numbers indicated on the right hand side of the lines represent five donors. **(D)** Summary of net A32-mediated cell lysis from six infections using PBMC from twelve donors as effector cells (each dot represents an individual). **(E and F)** Susceptibility of ∆Nef∆Vpu virus-infected cells to ADCC induced by A32 compared to that by 2G12. Shown in **(E)** is the result of a representative donor and in **(F)** is a summary of three experiments with seven donors, with each line representing data from a given individual. Statistical analysis of data was done using paired Student’s *t*-tests.

Using the established ADCC assay, we performed a comparative analysis of the susceptibility of T cells infected with the WT virus or its derivatives to ADCC. Prior to each analysis, as a qualitative control for the infection, we performed in parallel Env staining of target cells using appropriate test Ab(s). Shown in Figure 3A is an example of A32 staining for the ADCC analysis illustrated in Figure 3B. Although A32 recognized its epitope comparably well on T cells infected with ∆Nef or ∆Vpu virus, those infected with the ∆Nef appeared more susceptible to lysis than their ∆Vpu counterparts (Figure 3B). Intriguingly, T cells infected with the ∆Nef∆Vpu virus were consistently much more prone to lysis than all other target cells combined (Figure 3C and D). Also, the ADCC activity against the ∆Nef∆Vpu virus-infected targets was clearly not additive of that against ∆Nef and ∆Vpu virus. T cells infected with the WT virus were poorly susceptible to ADCC.

Given that ∆Nef∆Vpu virus-infected cells were invariably most prone to ADCC, we next compared the ability of A32 and 2G12 in activating killing of these target cells. For the example given in Figure 3E, the net Ab-specific cell lysis for A32 (60.2%-45.0% = 15.2%) was about 4-fold higher than that for 2G12 (48.7%-45.0% = 3.7%). Similar observations were made with PBMC from different donors (Figure 3F).

### A32-mediated ADCC activity is dependent on cell-surface CD4 expression and requires CD4-Env interaction

**Figure 4:**
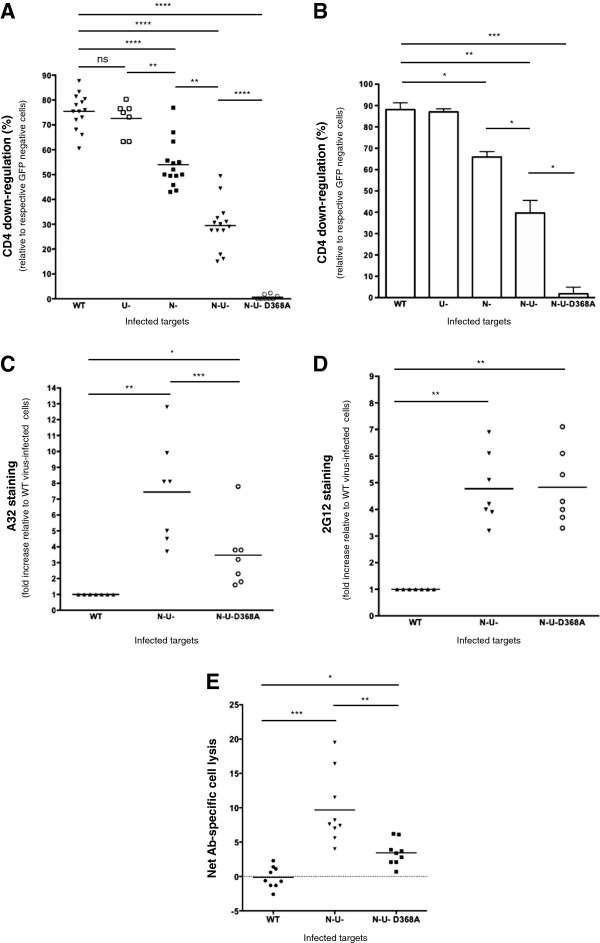
**Heightened susceptibility to A32-mediated ADCC is intimately dependent on CD4 expression and CD4-Env interactions on target cells.** CEM.NKR cells or primary CD4+ T cells were infected with CCR5-tropic NL4.3.ADA.IRES.GFP viruses as mentioned in Figure 1 legend. The ∆Nef∆Vpu-D368A Env (N-U-D368A) virus harbours a mutation within the CD4-binding site of Env protein leading to defective CD4-Env interactions. **(A-B)** CEM.NKR cells **(A)** and primary CD4+ T cells **(B)** were examined by flow cytometry for CD4 expression. The latter was determined based upon MFI values obtained for gated GFP^+^ cells. % CD4 down-regulation was calculated as: (MFI of infected cells / MFI of GFP^-^ (uninfected) cells) × 100. Shown are average % of CD4 down-regulation of **(A)** a series of experiments (each dot represents an analysis), or **(B)** five evaluations with five donors. Error bars indicate SEM. **(C-D)** CEM.NKR T cells infected with WT, ∆Nef∆Vpu or ∆Nef∆Vpu-D368A virus were evaluated for Env expression using A32 **(C)** and 2G12 **(D)** Abs as detailed in Figure 1 legend. Env staining was determined based upon the MFI values obtained for gated GFP^+^ cells. Calculations of fold increase in Env staining were as described in Figure 1 legend. **(E)** CEM.NKR T cells were evaluated for their susceptibility to A32-mediated ADCC as described in Figure 2 legend. Shown are average net cell lyses from four infections. Cell killing was done using PBMC from nine donors as effectors, with each dot representing an individual. Statistical analysis of data was done using paired Student’s *t*-tests.

At this point, our data have demonstrated a correlation between enhanced A32 binding and heightened ADCC susceptibility. This was most evident in T cells infected with the ∆Nef∆Vpu virus (Figures 1 and 3A-D). Given that the A32 epitope becomes transitionally exposed upon CD4-Env engagement during viral entry, and that both Nef and Vpu down-modulate CD4 within infected cells, we asked if the strength of ADCC function was related to CD4 expression. Indeed, CD4 down-modulation was greatest on WT-virus infected cells (about 75 and 90% on CEM.NKR [Figure 4A] and primary CD4+ T [Figure 4B] cells, respectively). Similarly, CD4 expression on ∆Nef and ∆Nef∆Vpu virus-infected cells was reduced by 50-60% and 25-35%, respectively, highlighting the individual contributions of Nef and Vpu to CD4 depletion. Nonetheless, the role of Vpu in CD4 down-regulation was less evident in the presence of Nef, owing presumably to the distinct mechanisms utilized by the two proteins to decrease CD4 expression [1,2]. Interestingly, the use of the ∆Nef∆Vpu-D368A Env virus, which harbours a mutation within the CD4-binding site of gp120 [28], completely prevented CD4 down-regulation, reaffirming the role of Env in interacting with CD4 and contributing to its depletion [3-5].

Interestingly, A32 staining on ∆Nef∆Vpu-D368A Env virus-infected T cells was significantly less (P < 0.005) compared to that on their ∆Nef∆Vpu virus counterparts (Figure 4C), highlighting the necessity of CD4-Env interactions within infected cells for unmasking the A32 epitope at the cell surface. In sharp contrast, 2G12 staining was not affected by the presence of the D368A mutation, suggesting that CD4-Env interactions are dispensable for 2G12 recognition (Figure 4D). Functionally, the reduction in A32 staining on ∆Nef∆Vpu-D368A Env virus-infected T cells was correlated with a statistically significant (P < 0.005) decrease in ADCC activity compared to that with the ∆Nef∆Vpu virus (Figure 4E).

### BST2 partially contributes to enhanced ADCC activity on infected T cells

**Figure 5:**
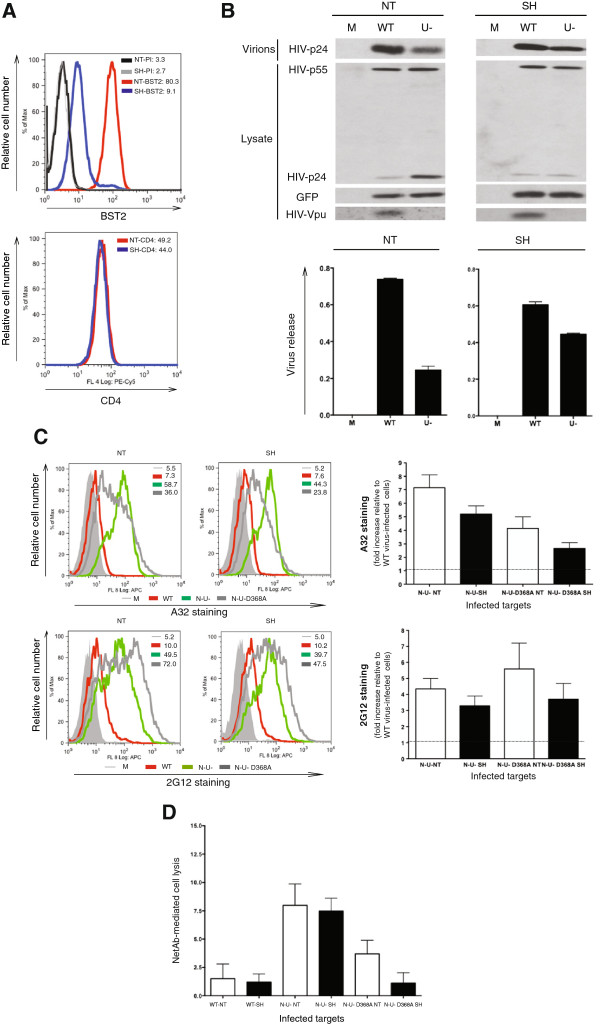
**Dissecting the potential involvement of BST2 in enhancing target cell lysis via ADCC.** CEM.NKR CD4+ T cells were transduced with a lentivirus containing a non-targeting (NT) or a BST2-targeting (SH) shRNA as described in Methods. **(A)** BST2 and CD4 expression on NT and SH cells as examined by flow cytometry. Parallel staining with a rabbit PI was used as a control for BST2 staining. Shown next to the overlays are expression levels in MFI obtained for gated GFP^+^ infected cells from a representative analysis. **(B)** NT and SH T cells were infected with WT or ∆Vpu (U-) virus and assessed for HIV-1 viral release efficiency by Western blotting. Mock (M)-infected cells were used as control. Parallel virions and cell lysates were analyzed for total Gag proteins, GFP and Vpu. The histograms underneath the Western blots depict the average quantifications of the densitometric signals from two experiments. Virus release was determined to be the ratio of the virion-associated Gag signal (corresponding to the mature p24) over all cell-associated Gag signals (corresponding to p24 and precursor p55). Within the NT and SH cells, viral release by WT virus was considered to be 100% and that by ∆Vpu virus counterparts was expressed as % of the WT. **(C-D)** NT and SH CD4+ T cells were infected with WT, ∆Nef∆Vpu (N-U-), or ∆Nef∆Vpu-D368A Env (N-U-D368A) virus. Env staining by A32 and 2G12 was analyzed by flow cytometry **(C)**. The histograms depict average fold increase (+/- SD) in Env recognition relative to WT virus-infected cells in three experiments. In parallel, target cells were evaluated for their susceptibility to A32-mediated cell lysis by ADCC as detailed in Figure 2 legend **(D)**. Shown are average (+/-SD) net cell lyses by PBMC from six donors. See also Additional file 1: Figure S1 and Additional file 2: Figure S2.

At this point, we clearly demonstrated the necessity of cell-surface CD4 accumulation and CD4-Env interactions for the A32 epitope to be exposed. However, the residual A32 staining and ADCC activity observed with the ∆Nef∆Vpu-D368A Env mutant, which were still higher than that seen with the WT virus (Figure 4C and E), implied a potential involvement of a CD4-independent factor. We thus hypothesized that the remaining 3-fold increase in A32 epitope recognition and ADCC activity was due to the accumulation of Env-containing virions at the cell surface arising from the absence of Vpu-mediated BST2 antagonism. To test this, we depleted BST2 from CEM.NKR T cells using a lentivirus-based vector that contains non-targeting (NT) or BST2-targeting (SH) shRNA. Using this system, BST2 expression was depleted in at least 95% of the cells (MFI 9.1 on SH cells *vs*. 80.3 on NT cells, Figure 5A upper panel), leaving about 5% still displaying BST2 at levels comparable to those on NT cells. Having said that, BST2 depletion in the 95% of cells was not complete since low-level BST2 was still detectable (compare staining of SH cells with pre-immune serum to that with BST2 Ab: MFI 2.7 *vs*. 9.1, respectively in Figure 5A). Of importance, the CD4 level was comparable between BST2-expressing (*i.e*., NT) and BST2-depleted (*i.e*., SH) cells. To confirm that BST2 depletion would mediate a change in virus particle release, the cells were infected with WT or ∆Vpu, (U-) virus, and evaluated for virus particle release (Figure 5B). As expected, the level of viral release by ∆Vpu virus-infected T cells in the NT cell line was about 25% of that from cells infected with the WT virus, reaffirming the role of BST2 in tethering virions at the cell surface (note the accumulation of mature p24 in cell lysates of ∆Vpu virus-infected T cells in the upper left panel of Figure 5B). In contrast, in BST2-depleted (SH) cells, the release of the Vpu-defective virus was restored to levels that were largely comparable to that of the WT virus (right panels, Figure 5B; in this context, cell-associated p24 levels were similar between WT and ∆Vpu virus-infected T cells). The observed partial restriction in viral particle release is likely the result of the incomplete BST2 depletion achieved with this cell line (Figure 5A).

Upon BST2 depletion, we found that Env recognition by the A32 and 2G12 Abs was differentially reduced in cells infected with the different viruses (Figure 5C and Additional file 1: Figure S1). Indeed, A32 staining on ∆Nef∆Vpu virus-infected T cells of the SH line was decreased by ~30-60% depending on experiments, while that on the ∆Nef∆Vpu-D368A Env counterparts, by ~50%. However, for both NT and SH cells, A32 binding on ∆Nef∆Vpu virus-infected T cells remained higher than that on cells infected with the ∆Nef∆Vpu-D368A Env virus, underscoring the independent contributions of CD4 and BST2 on A32 epitope exposure (Figure 5C, upper panels). In contrast, while 2G12 recognition was generally reduced upon BST2 depletion, the staining patterns between ∆Nef∆Vpu and ∆Nef∆Vpu-D368A Env virus-infected cells were similar for both cell lines. This finding reaffirmed the data shown in Figure 4D in that Env staining by 2G12 does not require CD4-Env interactions. Interestingly, in conditions where BST2 was depleted (Additional file 1: Figure S1) or naturally absent (Additional file 2: Figure S2), the patterns of A32 and 2G12 staining on ∆Vpu virus-infected cells were comparable to those on cells infected with the WT virus, implying that the enhanced Env recognition on BST2-expressing cells was likely attributed by the virion-tethering effect of BST2. On this note, analysis of Jurkat T cell lines expressing varying levels of CD4 and/or BST2 clearly revealed the relative contributions of CD4 and BST2 expression to the increase in Env staining by A32 and 2G12 in the context of WT and ∆Nef and/or ∆Vpu HIV infections (Additional file 2: Figure S2).

Functionally, we observed no significant decrease in ADCC activity against NT and SH cells infected with the ∆Nef∆Vpu virus (Figure 5D). However, the reduced A32 epitope exposure on ∆Nef∆Vpu-D368A Env virus-infected cells in the SH line abolished the “residual” ADCC activity, suggesting that BST2 most likely contributed to the ADCC function in ∆Nef∆Vpu-D368A Env virus-infected NT cells.

### Plasmas from HIV-infected individuals induced robust ADCC activity against T cells infected with the ∆Nef and ∆Vpu viruses

**Figure 6:**
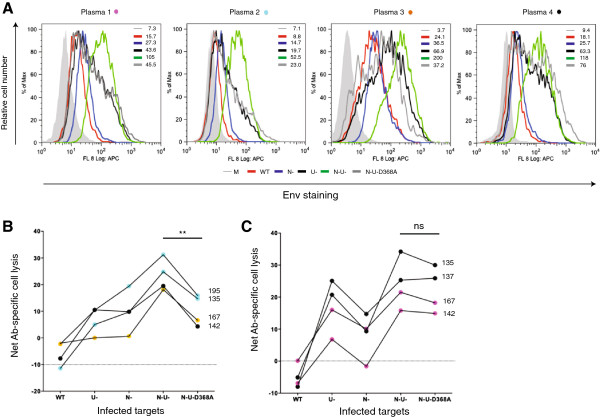
**The presence of Nef and Vpu efficiently shields HIV-infected T cells from ADCC mediated by plasma from HIV-infected individuals.** CEM.NKR cells were infected with viruses as mentioned in Figure 1 legend. **(A)** Target cells were stained with plasma samples from an HIV seronegative person (164HH) or from HIV-infected individuals (different plasmas were color coded). Env expression was determined as described in Figure 1 legend. **(B-C)** Target cells incubated with plasmas as described in **(A)** were analyzed for their susceptibility to ADCC using PBMC as effector cells. Net Ab-specific cell lysis was computed as described in Figure 2 and obtained following subtraction of background killing of the same targets induced by the 164 HH plasma. Panel **B** depicts ADCC activity mediated by patient plasmas, which appeared enriched with A32-like anti-Env Abs. Panel **C** illustrates ADCC activity induced by patient plasmas containing A32-like as well as other ADCC-competent anti-Env Abs. These characteristics of the patient plasmas were established post-analysis. The four sets of numbers indicated on the right hand side of the lines represent four donors. Data shown are representative of 3 analyses using plasmas from 6 infected individuals and PBMCs from 8 donors. Statistical analysis of data was done using paired Student’s *t*-tests.

To further validate the role of CD4 and BST2 in promoting ADCC in an *in vivo* relevant setting, we examined whether plasmas from HIV-infected individuals could mediate lysis of infected targets in a manner similar to A32. To this end, we found that the Env recognition patterns by patient plasmas nearly mirrored those by A32 (Figure 6A): the ∆Nef∆Vpu-virus-infected targets were most predominantly stained. As with A32, the use of the ∆Nef∆Vpu-D368A Env virus reduced the level of Env recognition, albeit to varying extent (1.5 to 5-fold) depending upon plasmas. In line with the A32 data, these infected plasmas elicited the most robust ADCC activity in ∆Nef∆Vpu-virus-infected targets (Figure 6B and C). Intriguingly, we found that for certain plasmas, the difference in ADCC activity against ∆Nef∆Vpu D368A Env and ∆Nef∆Vpu virus-infected targets was not statistically significant (compare N-U- to N-U-D368A of Figures 6B and 6C). Coincidentally, in the cases where this difference was not achieved (plasmas 1 and 4 shown in Figure 6C) we also observed a remarkably higher ADCC activity for the ∆Vpu relative to the ∆Nef virus implying perhaps a greater abundance of Abs behaving like 2G12 in these plasmas. It should however be mentioned that these characteristics of the plasmas were established post-analysis.

Aside from the differences in the types of anti-Env Abs that are potentially present, variations in host factors such as FcR expression levels and phenotype of the receptor may have also contributed to the kind of ADCC activity observed (compare plasma 4, donor 142 in Figure 6B to plasma 4, donors 135 and 137 in Figure 6C). This being said, these results clearly indicate that Vpu and Nef protect HIV-infected cells from ADCC-mediated by antibodies present in plasmas of HIV-infected individuals.

## Discussion

Recent studies have offered insights on the characteristics of the epitopes on HIV-1 gp120 that are recognized by ADCC-mediating Abs such as A32 [18,24]. With these results, there emerges a renewed interest in understanding not only how Abs targeting CD4-exposed epitopes could contribute to the overall HIV-induced ADCC response, but also how the virus might evolve to circumvent this mode of defence. Our data presented here-in demonstrate that enhanced exposure of the A32 epitope is intimately correlated with augmented ADCC activity against infected T cells. Importantly, the magnitude of epitope recognition by A32 is invariably dependent on both cell-surface expression of CD4 and Env and CD4-Env interactions on infected cells. Lastly, by removing CD4 and BST2 from the cell surface, HIV Nef and Vpu function individually yet synergistically to dampen infected cell susceptibility to ADCC. We postulate that it is these actions by the two accessory proteins that could help protect infected T cells from ADCC, revealing yet another mechanism of immune evasion exploited by HIV-1. In fact, the evidence reported here-in demonstrates that both Vpu and Nef contribute to the protection of HIV-infected cells from ADCC mediated by Abs that are present in plasmas of HIV-infected individuals. This underlies the *in vivo* relevance of our findings.

When CD4+ T cells were infected with the ∆Nef or the ∆Vpu virus, we observed a moderate increase of 2–3 fold in A32 binding compared to that on WT virus-infected T cells. Surprisingly, the A32 epitope recognition was significantly higher (8–16 fold) on T cells infected with the ∆Nef∆Vpu virus, suggesting a synergistic effect by Nef and Vpu. Functionally, this enhanced exposure of the A32 epitope led to heightened ADCC-mediated lysis of target cells, with those infected with the ∆Nef∆Vpu virus being most susceptible, and those infected with the ∆Nef virus moderately prone to ADCC. Subsequent analysis of CD4 expression at the surface of infected cells (WT, ∆Nef, ∆Vpu and ∆Nef∆Vpu) revealed a compelling inverse correlation between the extent of CD4 down-modulation and the degree of cell susceptibility to ADCC. Indeed, the most potently lysed ∆Nef∆Vpu virus-infected cells had only 30% of their CD4 down-regulated, while those moderately susceptible to ADCC (*e.g*., cells infected with the ∆Nef virus) had their CD4 expression reduced by 50%. In contrast, when the vast majority of CD4 was depleted from the cell surface, as in the case of WT or apparently ∆Vpu virus-infected cells, cells were poorly susceptible to ADCC. Having said that, the effect of Vpu on CD4 down-regulation may have been masked by that of Nef since Vpu’s activity was only evident in the context of the double mutant virus in primary CD4+ T cells and CEM NKR T cells. On this note, given the spatially separated and temporally distinct mechanisms exploited by Nef and Vpu to degrade CD4 [2], it is likely that in the presence of Nef, the effect of Vpu cannot be adequately quantified during the 48-hour infection. In turn, this may result in the seemingly lower susceptibility of ∆Vpu virus-infected targets to A32-mediated ADCC. One possible approach towards further elucidating the contribution of Nef-mediated down-regulation of cell-surface CD4 to ADCC would be to use Nef mutants that are incapable of mediating CD4 endocytosis. These evaluations, which are currently underway, would help corroborate our ADCC data with the ∆Nef virus. Since ADCC is mediated mainly by NK cells through the Fcγ receptor, it would seem unlikely that the Vpu-mediated downregulation of NK cell receptor SLAMF6/NTBA [29] may have contributed to the lower susceptibility of ∆Vpu virus-infected to ADCC.

The accumulation of CD4 at the cell surface was not sufficient to trigger cell lysis. We found that Env and CD4 interactions were necessary to unmask the A32 epitope since the use of the ∆Nef∆Vpu-D368A Env virus led to significantly reduced A32 binding and decreased ADCC function. In this context, similar findings were also recently reported by others [30]. On the notion of CD4-Env interactions, a recent analysis of cryo-EM structures of virion-associated Env trimer predicted the occlusion of the ADCC-competent JR4 epitope by gp41, in the presence of soluble CD4 (N. Gohain et al., 2013, Keystone Symposia: HIV vaccines, abstract). The cited study also revealed that JR4, together with other ADCC-competent Abs recognizing the so-called Cluster A epitopes [24], bound poorly to surface Env trimers in the presence of soluble CD4 but reacted efficiently with surface-bound virions, lending further support to the requirement of Env and cell-surface CD4 binding for the exposure of A32-like epitopes. Taken together, these findings suggest that CD4 molecules accumulated at the surface of infected cells would engage cell-surface Env molecules in a way similar to that when an incoming virion interacts with the CD4 receptor, through its Env, to enter a target cell.

Aside from showing the necessity of CD4-Env interactions, our analysis with the ∆Nef∆Vpu-D368A Env virus also implicated the involvement of BST2 in Env recognition, most likely through the retention of virus particles at the cell surface. Indeed, when BST2 was depleted at the plasma membrane, the residual ADCC activity against T cells infected with the ∆Nef∆Vpu-D368A Env was essentially abolished. As for the ∆Nef∆Vpu virus, although the depletion of BST2 led to a moderate reduction in A32 binding without significantly changing the ADCC function, we think this could have been due to the incomplete removal of BST2 from the CEM cells by the shRNA. BST2 contribution to the enhancementof ADCC function was strengthened by our findings with plasmas from HIV-infected individuals. They clearly show that BST2 can contribute significantly to this process. We are currently pursuing additional studies using Vpu mutants that are defective for BST2 binding to further strengthen this argument. These investigations would complement the analyses with the Nef mutants that were mentioned in the previous section and, as such, should provide key insights into the relative contributions of BST2 and CD4 to CD4-induced and non CD4-induced epitope-sensitization. At this juncture, however, the existing data strongly support the notion that CD4 is a more predominant player in potentially inducing a conformational change in the Env and, as such, a more pronounced ADCC function. On a relevant note, a recent work has postulated a role for ADCC in the complete protection of macaques previously vaccinated with a unique live attenuated SIV [17]. They revealed that animals inoculated with a persistent SIV∆*nef* strain mounted potent ADCC activity, and that it was this Ab-mediated effector response that afforded the apparent sterilizing protection against SIV_mac_251 challenge. In this context, our findings that CD4 and BST2 contributed to ADCC enhancement warrant further investigations since in SIV, Nef down-regulates both BST2 and CD4 [31,32].

Since 2G12 recognizes mannose residues on the exterior domain of gp120 [27], the residual binding of this Ab on BST2-depleted cells infected with the ∆Nef∆Vpu-D368A Env virus could be an accumulative consequence of carbohydrate modulations by Vpu and/or Nef and of virus particle retention by the residual BST2 that remained at the cell surface. In any case, regardless of how the 2G12 epitope might be recognized on T cells infected with the viruses used in our study, the key message is that 2G12 binding is not dependent on CD4-Env interactions, and that despite its efficient binding on Env, 2G12 is still significantly less potent than A32 at inducing ADCC. This observation was consistent with those reported earlier using CEM.NKR_CCR5_ T cells coated with recombinant Env or infected with HIV [18].

## Conclusions

In summary, our study provides a further insight as to how epitopes recognized by ADCC-mediating Abs can be most efficiently accessible on target cells. This work also unveils yet another mechanism by which HIV, through its accessory proteins Nef and Vpu, can evade the host’s immune defenses. Hence, by allowing for efficient release of progeny virus particles and preventing CD4 accumulation at the cell surface, HIV-1 ensures that Env epitopes targeted by ADCC remain unexposed. Therefore, inhibition of Vpu and Nef, could represent a promising therapeutic avenue to render infected cells susceptible to ADCC.

## Methods

### Reagents and antibodies

Phytohemagglutinin-L was purchased from Sigma-Aldrich (St. Louis, MO, USA). The eFlour670 dye was from eBioscience. Human recombinant interleukin-2 (IL-2) [33] was obtained through the NIH AIDS Research and Reference Reagent Program. Anti-GFP was acquired from Invitrogen. Mouse anti-p24 mAb (Cat. # HB9725) was isolated from the culture supernatant of hybridoma cells from the American Type Culture Collection. PerCP-Cy 5.5-conjugated anti-human CD4, allophycocyanin (APC)-conjugated anti-human Fc Ab, and AF647-conjugated anti-IgG secondary Ab were from Biolegend. Anti-HIV gp120 mAbs A32 and 2G12 were obtained through the NIH AIDS Research and Reference Reagent Program from Dr. James E. Robinson [34] and Dr. Hermann Katinger [27], respectively. The A32 Fab was obtained from Dr. Guido Ferrari and Dr. Barton Haynes (Center for HIV-AIDS Vaccine Immunology, Duke University). Anti-Vpu and anti-BST2 rabbit sera were described previously [35]. HIV-infected plasmas were obtained through the Montreal Primary Infection cohort that is part of the Fonds de Recherche du Québec-Santé (FRQ-S) AIDS Network. Plasmas from HIV- and HCV- seronegative donors were obtained through a cohort of healthy volunteers maintained at the Institut de recherches cliniques de Montréal (IRCM). Research protocols were approved by the research ethics review board at the IRCM.

### Plasmid and proviral DNA constructs

The vesicular stomatitis virus (VSV) glycoprotein G-expressing plasmid, pSVCMVin-VSV-G, was previously described [35].

The infectious CCR5-tropic NL4.3.ADA.IRES.GFP wild-type (WT), which contains all accessory proteins, and its Vpu-deficient derivative (∆Vpu, U-) were generated as described [36]. The WT construct was used to generate various isogenic proviruses using standard molecular biology techniques. The NL4.3.ADA.IRES.GFP∆Nef (N-) was created by introducing a frame-shift mutation at the unique Xho1 site within the Nef coding region, thus, generating a truncated inactive Nef protein of 38 amino-acid residues. The isogenic NL4.3.ADA.IRES.GFP ∆Nef∆Vpu (N-U-) combined the two mutations present in the ∆Vpu and ∆Nef proviral constructs, while the NL4.3.ADA.IRES.GFP ∆Nef∆Vpu-D368A Env (N-U-D368A) was generated by introducing a substitution mutation (D/A) at position 368 of Env using Quickchange mutagenesis (Stratagene).

### Production of VSVg-pseudotyped lentiviral vectors and HIV-1 viruses

For lentiviral vector production, HEK293 T cells were transfected with plasmid pLKO.1 (puromycin-resistant) expressing shRNA targeting BST2 (Clone ID: TRCN0000107018, from OpenBiosystem) or control shRNA together with the packaging construct psPAX2 (a gift from Dr. D. Trono at Swiss Institute of Technology) and VSV-G expressing plasmid pSVCMVin-VSV-G using a calcium phosphate precipitation method. Vectors were purified by ultracentrifugation 48 h later [37].

For virus production, HEK 293 T cells were transfected with appropriate proviruses and pSVCMVin-VSV-G using the calcium phosphate method [35]. Viruses were harvested 48 h later [37] and titrated by MAGI assay as described [38].

### Preparation of CEM.NKR CD4+ T cell line depleted of BST2

CEM.NKR cells were transduced by spin-inoculation [39] using lentiviral vector particles containing shRNA targeting BST2 or control shRNA. Forty-eight hours later, puromycin was added and puromycin-resistant cells were selected after 10 days. BST2 expression was determined by flow cytometry. CEM.NKR T cells expressing or depleted of BST2 were characterized functionally for viral release 48 h post-infection with the CCR5-tropic NL4-3.ADA.IRES.GFP WT or NL4-3.ADA.IRES.GFP∆Vpu virus.

### Jurkat T cell lines: phenotype, infection and assessment of surface molecule expression by flow cytometry

Four different Jurkat T cell lines were used in this study. Their phenotype for CD4 and BST2 expression as determined by flow cytometry is as follows. The first line, derived from a Jurkat line that stably expresses the SV40 large T antigen [40], is negative for both CD4 and BST2 [41] and shall be referred to in the paper as CD4-/BST2-. The second line, a derivative of the first one, expresses high levels of CD4 upon stable transfection with a SV40 origin-containing CD4 expressor. This line is referred to in the paper as CD4hi/BST2-. The third and fourth lines of Jurkat are derivatives of the E6.1 clone from the ATCC. The former expresses minimally CD4 and positive for BST2 (CD4lo/BST2), while the latter expresses CD4 and BST2 at relatively comparable levels (CD4+ /BST2+).

### Preparation of primary effector/target cells and infection of CD4+ T cells

Peripheral blood mononuclear cells (PBMC) were prepared from whole blood of HIV- and HCV- seronegative donors as described [39]. In certain experiments where effector cells were destined to have no monocytes/macrophages, PBMC were cultured in serum-free RPMI 1640 (Wisent) for 2 h to remove monocytes/macrophages by plastic-adherence. Non-adherent cells were recovered to be used as effector cells. When effector cells were to contain no NK cells, PBMC were depleted of NK cells using CD56 microbeads (Miltenyi Biotec). Cell purity was confirmed by flow-cytometry using anti-human CD14 or anti-human CD56 Abs, respectively. In all cases, effector cells to be used in ADCC assays were cultured overnight in complete RPMI 1640 medium (10% FBS supplemented with L-Glutamine, Pennicilin-streptomycin, and 100 U/mL IL-2).

For HIV-infection of CD4+ T cells, activated primary CD4+ T cells were spin-infected [39] as well as T cell lines (CEM.NKR and Jurkat) with CCR5-tropic NL4.3.ADA.IRES.GFP viruses at multiplicity of infection of 0.5-1 depending on cell types. Forty-eight hours post-infection, T cells were analyzed by flow cytometry, when appropriate, for CD4, BST2 and Env expression or for ADCC activity.

### Flow cytometry

CD4 staining was done as per manufacturer’s protocols. For Env staining, infected cells were stained with anti-human Env primary Abs (A32 or 2G12) or control IgG for 30 min at 4°C, and then exposed to APC-conjugated anti-human Fc secondary Ab (Biolegend). For Env staining analyses using human plasmas, target cells were stained with diluted plasma (1:250 to 1:1000 dilutions) from HIV-infected individuals or as control, with plasma from healthy donors. Staining conditions were as described for A32. Fluorescence signals were revealed using AF647-conjugated anti-IgG secondary Ab. In certain experiments where A32 binding specificity was being evaluated, cells were pre-exposed to the A32 Fab (3.8 μg/mL) for 30 min at room temperature (RT) prior to the A32 incubation step.

BST2 staining was done as described using anti-rabbit BST2 Ab or as a control, a rabbit pre-immune (PI) serum [35].

### Antibody-dependent cytotoxicity assay (ADCC)

50,000 target (T) cells, plated in 96-well V-bottom plates, were exposed to A32 (1.4 μg/mL), 2G12 (1.4 μg/mL) or human control IgG Ab for 30 min at RT. In certain experiments, target cells were pre-incubated with the A32 Fab for 30 min before adding A32. As an alternative source of Abs, target cells were incubated with empirically-determined concentrations of plasmas (1:250–1:1000 dilutions) from HIV-infected patients or from healthy donors (as control). Effector cells, that had been labelled with eFlour670 dye were mixed with target cells at effector:target ratios of 10:1 to 30:1. The cell mixtures were spun for 3 min at 400 × *g* and cultured for 4–4.5 h at 37°C. Subsequently, medium was removed by centrifugation, cells were fixed in 1% PFA and analyzed on a CyAn ADP analyzer for GFP expression. Percent of cell lysis was determined as [(% GFP^+^ in the absence of effectors -% GFP^+^ in the presence of effector cells and test Ab)/% GFP^+^ in the absence of effectors] × 100. Depending on analysis, test Ab could be plasma (uninfected or infected), IgG, 2G12, A32 or A32 Fab + A32.

### Viral particle release assay

BST2-expressing or BST2-depleted CEM.NKR CD4+ T cells were infected for 48 h with CCR5-tropic NL4-3.ADA.IRES.GFP WT or ∆Vpu virus. Virions were purified by ultracentrifugation [35]. Virions and cells were lysed in RIPA-DOC buffer (10 mM Tris pH 7.2, 140 mM NaCl, 8 mM Na_2_HPO_4_, 2 mM NaH_2_PO_4_, 1% Nonidet-P40, 0.5% sodium dodecyl sulfate, 1.2 mM deoxycholate), and Western blotting was performed [35] using Abs specific for Vpu, GFP or Gag (the anti-Gag Ab recognizes the precursor p55 and the processed forms of Gag, including p24).

### Statistical analyses

Unless otherwise stated, data are expressed as average ± SEM. Statistical analyses of the data were done using two-tailed, paired (when appropriate) Student’s *t*-tests. P values of ≤ 0.05 were considered statistically significant: * ≤ 0.05; ** ≤ 0.005; ***≤0.0005; ****≤0.00005; and ns, not significant.

## Competing interests

The authors declare that they have no competing interests.

## Authors’ contributions

TNQP, SL and EAC conceived and designed experiments. TNQP, SL and FH performed the experiments. J-PR provided patient plasmas. TNQP, SL and EAC analyzed the data. TNQP and EAC wrote the manuscript. All authors read and approved the final manuscript.

## Supplementary Material

Additional file 1: Figure S1 Effect of BST2 depletion on HIV-1 envelope expression profiles on infected CD4+ T cells. BST2 was depleted from CEM.NKR CD4+ T cells as described in Methods. BST2-expressing (NT) and BST2-depleted (SH) CEM.NKR T cells were infected with CCR5-tropic NL4.3.ADA.IRES.GFP WT virus or derivatives lacking Vpu (U-), Nef (N-) or both (N-U-). The N-U- D368A viral construct contains a mutation at residue 368 of Env which prevents CD4-Env interactions. Forty-eight hours post-infection, cells were stained with A32 (A) or 2G12 (B) Abs and analyzed for Env expression by flow cytometry. Mock (M)-infected cells stained in parallel were used as control. Shown next to the overlays are Env levels in MFI obtained for gated GFP + infected cells from a representative analysis. The histograms shown depict the average fold increase (+/- SD) in Env staining relative to WT virus-infected cells in two experiments. Click here for file

Additional file 2: Figure S2 Examining the relative contributions of CD4 and BST2 in promoting Env staining by A32 or 2G12 Abs. Jurkat T cell lines which vary in their expression of CD4 and BST2 (A) were infected with NL4.3.ADA.IRES.GFP WT virus or derivatives lacking Vpu (U-), Nef (N-) or both (N-U-) as described in Methods. Forty-eight hours later, cells were stained for (B) CD4 and BST2 and for Env using (C) A32 or (D) 2G12 Abs, and analyzed for their expression by flow cytometry. Mock (M)- infected cells stained in parallel were used as control. Indicated next to the overlays (A, C and D) were expression levels shown in MFI for infected T cells (GFP+) from a representative analysis. The histograms (B) depict the percentage of CD4 or BST2 down-regulation in GFP-positive cells relative to respective GFP-negative cells. Click here for file
