# Supplementary figures and images for: HIV Nef and Vpu protect HIV-infected CD4+ T cells from antibody-mediated cell lysis through down-modulation of CD4 and BST2
Source: Retrovirology. 2014 Feb 6;11:15. doi: 10.1186/1742-4690-11-15 (PMC3930549; doi:10.1186/1742-4690-11-15)

A

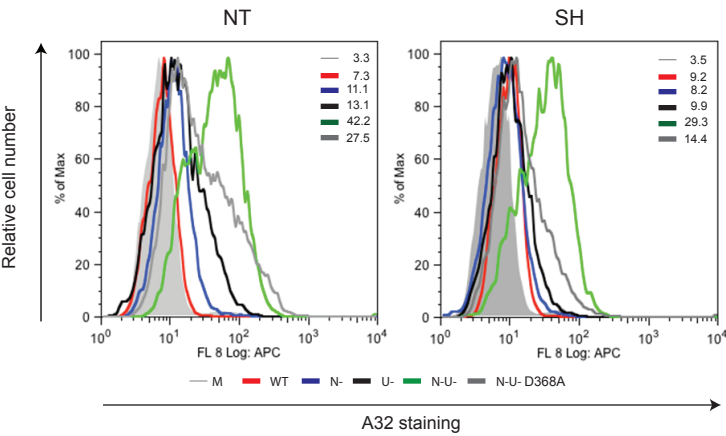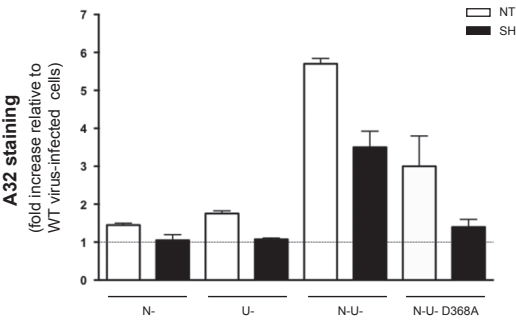

B

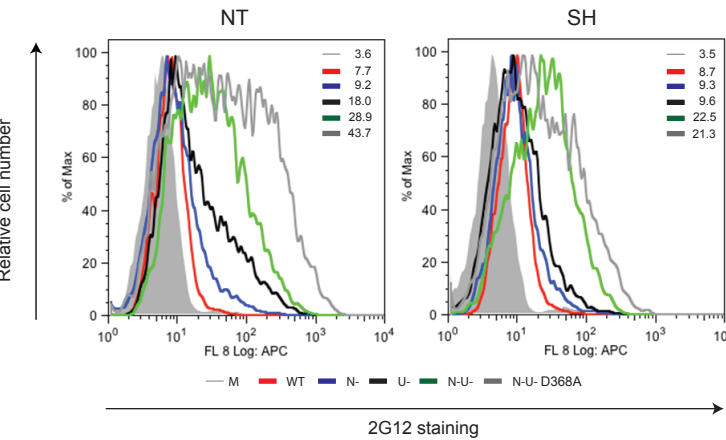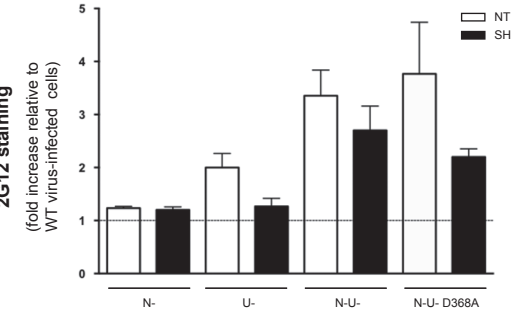

Supplement: Additional file 1: Figure S1 — Effect of BST2 depletion on HIV-1 envelope expression profiles on infected CD4+ T cells. BST2 was depleted from CEM.NKR CD4+ T cells as described in Methods. BST2-expressing (NT) and BST2-depleted (SH) CEM.NKR T cells were infected with CCR5-tropic NL4.3.ADA.IRES.GFP WT virus or derivatives lacking Vpu (U-), Nef (N-) or both (N-U-). The N-U- D368A viral construct contains a mutation at residue 368 of Env which prevents CD4-Env interactions. Forty-eight hours post-infection, cells were stained with A32 (A) or 2G12 (B) Abs and analyzed for Env expression by flow cytometry. Mock (M)-infected cells stained in parallel were used as control. Shown next to the overlays are Env levels in MFI obtained for gated GFP + infected cells from a representative analysis. The histograms shown depict the average fold increase (+/- SD) in Env staining relative to WT virus-infected cells in two experiments. [file 1742-4690-11-15-S1.pdf]

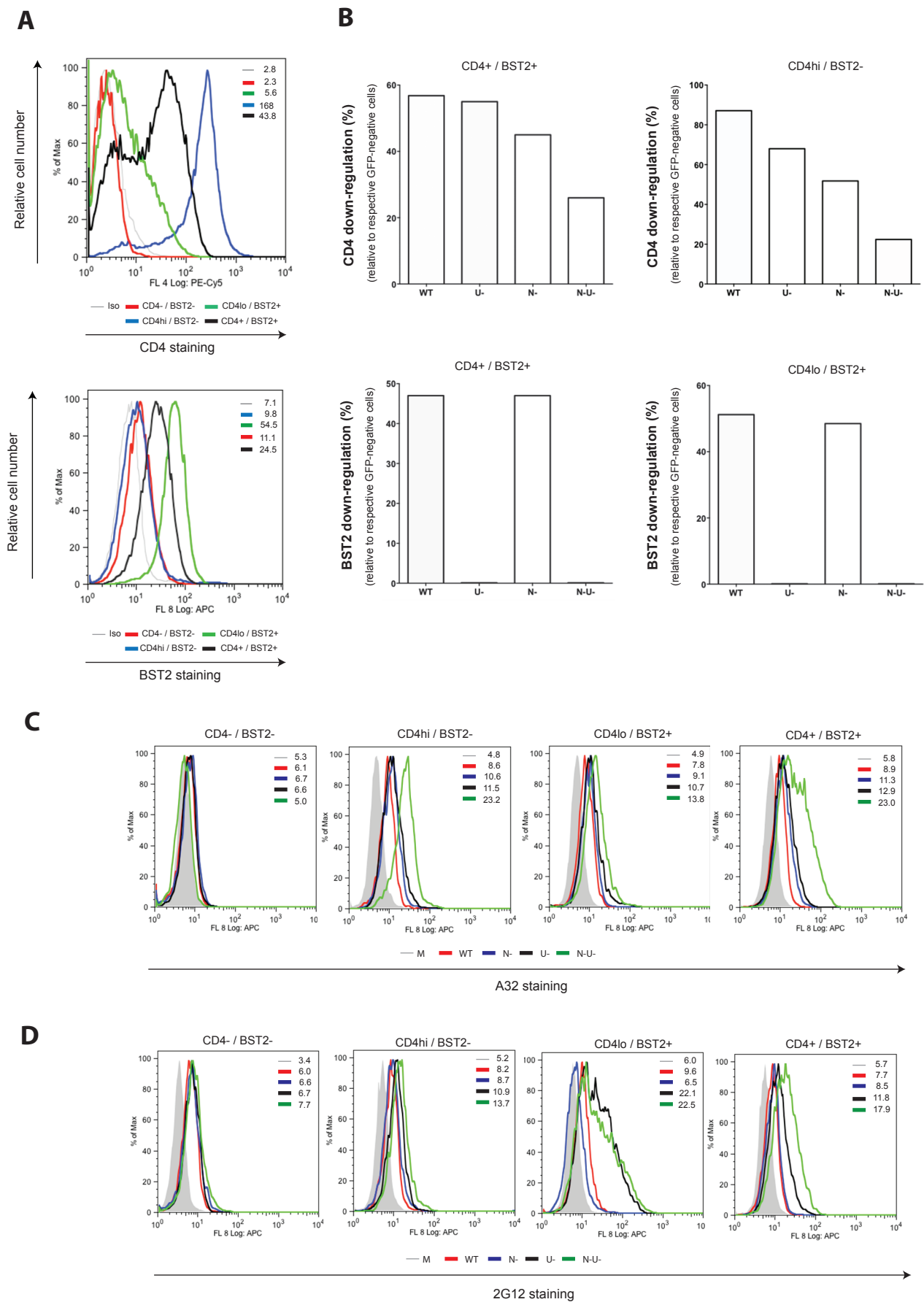

Additional file 2: Figure S2

Supplement: Additional file 2: Figure S2 — Examining the relative contributions of CD4 and BST2 in promoting Env staining by A32 or 2G12 Abs. Jurkat T cell lines which vary in their expression of CD4 and BST2 (A) were infected with NL4.3.ADA.IRES.GFP WT virus or derivatives lacking Vpu (U-), Nef (N-) or both (N-U-) as described in Methods. Forty-eight hours later, cells were stained for (B) CD4 and BST2 and for Env using (C) A32 or (D) 2G12 Abs, and analyzed for their expression by flow cytometry. Mock (M)- infected cells stained in parallel were used as control. Indicated next to the overlays (A, C and D) were expression levels shown in MFI for infected T cells (GFP+) from a representative analysis. The histograms (B) depict the percentage of CD4 or BST2 down-regulation in GFP-positive cells relative to respective GFP-negative cells. [file 1742-4690-11-15-S2.pdf]
